# Aberrant development of intrinsic brain activity in a rat model of caregiver maltreatment of offspring

**DOI:** 10.1038/tp.2016.276

**Published:** 2017-01-17

**Authors:** C-G Yan, M Rincón-Cortés, C Raineki, E Sarro, S Colcombe, D N Guilfoyle, Z Yang, S Gerum, B B Biswal, M P Milham, R M Sullivan, F X Castellanos

**Affiliations:** 1 CAS Key Laboratory of Behavioral Science, Institute of Psychology, Beijing, China; 2 Magnetic Resonance Imaging Research Center, Institute of Psychology, Chinese Academy of Sciences, Beijing, China; 3 Department of Child and Adolescent Psychiatry, NYU Langone Medical Center School of Medicine, New York, NY, USA; 4 Center for Biomedical Imaging and Neuromodulation, Nathan Kline Institute for Psychiatric Research, Orangeburg, NY, USA; 5 The Emotional Brain Institute, Nathan Kline Institute for Psychiatric Research, Orangeburg, NY, USA; 6 Center for the Developing Brain, Child Mind Institute, New York, NY, USA; 7 Department of Biomedical Engineering, New Jersey Institute of Technology, Newark, NJ, USA

## Abstract

Caregiver maltreatment induces vulnerability to later-life psychopathology. Clinical and preclinical evidence suggest changes in prefrontal and limbic circuitry underlie this susceptibility. We examined this question using a rat model of maternal maltreatment and methods translated from humans, resting-state functional magnetic resonance imaging (R-fMRI). Rat pups were reared by mothers provided with insufficient or abundant bedding for nest building from postnatal (PN) days 8 to 12 and underwent behavioral assessments of affect-related behaviors (forced swim, sucrose preference and social interaction) in adolescence (PN45) and early adulthood (PN60). R-fMRI sessions were conducted under light anesthesia at both ages. Offspring reared with insufficient bedding (that is, maltreated) displayed enduring negative affective behaviors. Amygdala-prefrontal cortex (PFC) functional connectivity increased significantly from adolescence to adulthood in controls, but not in maltreated animals. We computed the fractional amplitude of low-frequency fluctuations (fALFF), an index of intrinsic brain activity, and found that fALFF in medial prefrontal cortex and anterior cingulate cortex (MPFC/ACC) increased significantly with age in controls but remained unchanged in maltreated animals during adolescence and adulthood. We used a seed-based analysis to explore changes in functional connectivity between this region and the whole brain. Compared with controls, maltreated animals demonstrated reduced functional connectivity between MPFC/ACC and left caudate/putamen across both ages. Functional connectivity between MPFC/ACC and right caudate/putamen showed a group by age interaction: decreased in controls but increased in maltreated animals. These data suggest that maltreatment induces vulnerability to psychopathology and is associated with differential developmental trajectories of prefrontal and subcortical circuits underlying affect regulation.

## Introduction

Early-life experiences shape brain development and enable environmental inputs to interact with genetic factors to permit neurobehavioral adaptation to diverse environments. However, this open system for brain development entails risk, as harsh environments, such as caregiver maltreatment, can disrupt typical brain development and initiate a pathway to pathology.^1, 2^ Indeed, infant maltreatment, especially from the caregiver, is associated with aberrant brain development, compromised affect regulation and later-life psychiatric disorders.^3, 4, 5, 6, 7, 8^ The effects of maltreatment preferentially target developing emotion circuits in both animals^9, 10, 11, 12^ and humans,^13, 14, 15, 16^ along with ubiquitous brain effects.^3, 17^ Brain structures exhibiting prolonged maturation, such as the amygdala and prefrontal cortex, are particularly sensitive to environmental influences,^3^ including early rearing conditions and stressors that disrupt mother–infant interactions.^1, 9, 12, 18^ Overall, there is convergence in the human and animal literatures concerning the impact of early-life adversity, although different approaches and techniques across species hinder translation.^19^

The amygdala and the prefrontal cortex (PFC) are implicated in emotion reactivity and regulation,^20, 21, 22^ respectively, and later-life brain and behavioral outcomes associated with maltreatment.^3, 23, 24, 25, 26^ Animal models suggest a causal relationship,^9, 27^ although the expression of these neurobehavioral effects is frequently delayed.^28, 29^ Furthermore, although recent human research suggests that functional connectivity between the amygdala and PFC is also compromised following early-life stress involving disruptions in typical mother–infant relationships (trauma, deprivation and orphanage rearing),^13, 30, 31, 32^ the developmental trajectory of amygdala and PFC functional connectivity in animal models is less clear. To assess this, we used resting-state functional magnetic resonance imaging (R-fMRI),^33, 34^ a widely used tool for mapping human brain networks, to question how PFC-amygdala functional connectivity might change due to early-life trauma (that is, caregiver maltreatment) and brain maturation across two ages (adolescence and young adulthood). This approach begins to address the time course of trauma-induced changes in the developing brain.

To this end, rat pups were reared by a mother provided with insufficient bedding for nest building (maltreatment group) or by one with abundant bedding (controls) during infancy. In adolescence and adulthood, animals were used for either two R-fMRI sessions or for behavioral assessments of affect-related behaviors (forced swim, sucrose preference and social interaction). On the basis of demonstrations of alterations in amygdala and PFC due to disruptions in maternal care in nonhuman primates and rodents,^9, 11, 12, 35, 36, 37, 38, 39, 40, 41^ we hypothesized maltreated rats would show differential intrinsic functional connectivity between amygdala and PFC during development. As the effects of early-life trauma are ubiquitous in the brain, beyond functional connectivity analysis, we also examined changes in the strength of intrinsic brain activity throughout the brain.

## Materials and methods

### Animals

One hundred and twenty-five male Long Evans rats (behavior *n*=75; fMRI *n*=50) were born in our vivarium. Dams and pups were housed in polypropylene cages (34 × 29 × 17 cm) lined with abundant pine shavings, *ad libitum* food and water, and kept in a temperature (23 °C) and light (from 0700 to 1900 hours) controlled room. The day of parturition was considered postnatal day 0 (PN0) and litters were culled to 12 pups on PN0-1. At PN8, litters were assigned to one of two rearing conditions (see below) in a randomized manner. Approval was received for all procedures from Institutional Animal Care and Use Committee of the Nathan Kline Institute for Psychiatric Research, which followed National Institutes of Health guidelines. For behavioral assessments, animals were only used once to avoid effects of learning and stress during the previous test on later task performance. Sample sizes were selected according to our previously published work^9, 12, 42^ and all behavioral experiments were run blind to experimental condition. For imaging, animals raised under identical conditions were each scanned twice: first in adolescence (~PN45, adolescence), and then again in young adulthood (~PN60, adults).

### Naturalistic stressed-mother paradigm

Mother and pups were housed with either limited (1000 ml, 1.2 cm layer) or abundant (4500 ml, 5 cm layer) nesting/bedding material from PN8-12. This limited bedding environment decreases the mothers’ ability to construct a nest and results in frequent nest building, more time away from pups, rough handling and stepping on pups and less maternal care.^9, 37, 43^ Under this condition, pups nurse less and have elevated corticosterone levels, although weight gain is normal.^9, 37, 44, 45, 46^ Maternal and pup behaviors were recorded twice a day in 30 min sessions. Stepping on pups, rough handling (that is, mother aggressively grooms pups, transporting pups by limb), pup vocalization, mother’s time in the nest and nursing were quantified, as documented previously.^9, 37, 46^

### Behavioral testing

#### Sucrose preference test

For the sucrose preference test, rats (adolescence: 5 maltreated and 6 controls; adults: *n*=6 per condition) were acclimated for 36 hr and given access to a 1% (w/v) sucrose/water solution or tap water in the home cage to avoid neophobia during testing.^47^ Bottle positions were switched two to three times to prevent the formation of location preference. Before the sucrose preference test, animals were deprived of water for ~3 h and then placed in a new cage for testing. The test began with two bottles, each containing tap water or the sweet solution. After 60 min, fluid intake was measured and sucrose preference was calculated as the difference in bottle weight (g) of sucrose solution ingested.

#### Forced swim test

For the forced swim test, we used the original version^48^ previously used in our laboratory.^9, 47, 49^ A total of 24 animals were tested (Maltreatment, *n*=4; Control, *n*=8 for both adolescence and adulthood). Rats were placed in a plastic cylinder (*d*=25cm; *h*=65 cm; water temperature 28±1 °C) large enough so that the tail did not touch the base. On day 1, animals were given a 15 min pretest swim. On day 2, rats were administered a 5 min swim test and the latency to immobility was recorded. Following both sessions, animals were gently dried, placed in a 32 °C chamber and returned to the home cage. Water was changed between animals.

#### Social interaction test

Animals were tested in a two-chamber apparatus (61 × 60 × 61 cm) built of black Plexiglas sheets for the sides and a white bottom to form an open top box. A black Plexiglas division (60.5 × 61 cm) separated the two chambers and a (15 × 13 cm) square opening allowed animals to move between chambers. Two metal cubes (15 × 15 × 30 cm), with circular holes (1 cm) placed 1 cm apart on each of the 4 sides, were placed in the chambers (1 per chamber) during a 5-min habituation period; time spent by animals in each chamber during habituation was recorded. After habituation, a younger same sex animal (to prevent aggressive or mating behaviors) was placed inside one of the metal cubes as a social stimulus. Time spent in each chamber (social vs nonsocial) was recorded for 10 min. Animals were tested either in adolescence (PN45-55; 7 rats per condition) or adulthood (PN70-80; 6 in the maltreatment and 8 in the control condition).

### Resting-state functional MRI

#### Data acquisition

We scanned 50 rats (Maltreatment, *n*=25; Control, *n*=25) twice: at adolescence (Controls: PN 45.3±0.98 (s.d.); Maltreated: PN 45.5±1.16), and again in early adulthood (Controls: PN 60.0±1.06; Maltreated: PN 59.9±1.36). The groups did not differ in age at either scan or in inter-scan interval. Image acquisition consisted of interleaved snapshot echo planar imaging. This approach splits the conventional echo planar imaging sequence into a series of excitation-acquisition blocks applied in immediate succession within a single repetition period.^50^ All data were obtained on a 7.0 T Agilent (Santa Clara, CA, USA) 40 cm bore system. The gradient coil insert has an internal diameter of 12 cm with a maximum gradient strength of 600 mT/m and minimum rise time of 200 μs. A Rapid (Rimpar, Germany) volume-transmit (72 mm ID) and a 4-channel receive-only surface coil was used with the following parameters: field of view=40 mm (64 × 64), slice thickness=1 mm, 14 slices with a 0.1 mm gap, echo time=20 ms, repetition time=2 s, number of volumes=360, 3 segments with variable flip angles=35°, 45° and 90°.^50^ Five 12-min runs were obtained per rat per session with constant 1.5% isoflurane anesthesia.

A proton density weighted fast spin echo imaging method was used for anatomical scans with the same localization parameters. An SA Instruments animal monitoring unit (model 1025, Stony Brook, NY, USA) was used to monitor respiration, heart rate and rectal temperature. Respiration was measured with a pressure transducer placed under the abdomen just below the ribcage. An infrared pulse oximeter sensor, placed on the tail or hind foot, enabled heart rate monitoring. Body temperature was maintained using forced warm air, controlled by a feedback circuit between the heater and thermistor. All animals were anesthetized using an isoflurane vaporizer set at: 3–4% for induction, 2% during piloting, and 1.5% during scanning. After induction, subjects were placed on the RF coil tray and head motion restrained using a bite bar. Oxygen was used as the carrier gas and delivered at low flow rate (⩽ 0.5 l min^−1^) to a cone positioned before the bite bar, where gases mixed with air were passed over the rodent’s nose. All animals were maintained at 37±0.2 °C.

#### Preprocessing

Preprocessing was performed using the Data Processing Assistant for Resting-State fMRI (DPARSF^51^) V4.0 Rat module, which is based on Statistical Parametric Mapping (SPM8; www.fil.ion.ucl.ac.uk/spm) and the toolbox for Data Processing & Analysis of Brain Imaging (DPABI,^52^ rfmri.org/DPABI). Briefly, the voxel dimensions of both anatomical and echo planar imaging images were scaled by a constant factor of 10 to optimize their utilization in standard neuroimaging software packages designed for human imaging. A group-specific template was created based on a Paxinos rat brain template^53^ with the following steps: (1) the anatomical image of each rat at each scan was linearly coregistered to the rat brain template; (2) the coregistered images were averaged into an intermediate group template; (3) each anatomical image was non-linearly normalized to the intermediate group template, and then averaged to a group-specific template. Each individual was normalized to the group-specific template, while applying the parameters to the functional images. This utilization of the group-specific template is effective in minimizing systematic coregistration errors in situations in which morphological differences (for example, age at scan) might bias spatial coregistration to a standard template.^54^

After slice-timing correction, motion correction and spatial normalization, the echo planar imaging images were smoothed with a full width-half maximum kernel at 0.6 mm. Six head motion parameters were regressed out from the functional data to minimize influence of nuisance covariates. Of note, as the rats were anesthetized, the head motion was minimal as measured by framewise displacement (using the Jenkinson definition,^55^ modifying the assumed head radius from 80 mm to 8 mm): at adolescence (Controls: 7.3±2.9 μm; Maltreated: 6.5±2 μm), and again in early adulthood (Controls: 7.2±3.1 μm; Maltreated: 7.2±2.3 μm). The groups did not differ in head motion at either scan or across scans. Global signal regression was not performed because of controversy regarding increasing negative correlations^56, 57^ and possibly distorting group differences in functional connectivity.^58, 59^ Functional data were then subjected to a 0.01–0.1 Hz band-pass filter.

#### Hypothesis-driven analysis

To examine functional connectivity between amygdala and PFC, we divided the amygdala into basolateral, lateral and central parts according to the Paxinos atlas.^53^ For PFC, we defined 3 regions-of-interest (ROIs): prelimbic (PL); infralimbic (IL); and anterior cingulate cortex (ACC). We extracted mean time series from the 3 amygdala ROIs and 3 PFC ROIs, and calculated the correlations for the 9 amygdala–PFC pairs. For each rat, correlations were first averaged across the five sessions. The overall correlations for each animal at each of the two ages scanned (PN45 and PN60) were then forwarded to a mixed-effects analysis of variance (ANOVA), with age modeled as a within-subjects factor, and treatment group (Maltreatment vs Control) as a between-subjects factor. The interaction between age and treatment group was modeled as the effect of interest. The nine comparisons (nine pairs) were subjected to false discovery rate correction.

#### Exploratory whole-brain analysis

For each animal and scan, we computed the fractional amplitude of low-frequency fluctuations (fALFF)^60^ based on the preprocessed data without band-pass filtering. The fALFF is the ratio between the sum of amplitudes within a specific low-frequency range (0.01–0.1 Hz) and the sum of Fourier amplitudes across the entire frequency range, thus representing the relative contribution of specific oscillations to the entire detectable frequency range. To further evaluate the whole-brain functional connectivity of those clusters demonstrating significant group differences or group by age interaction, Pearson’s correlation coefficients were calculated between each voxel time series and each of the significant clusters, and then converted to *z* values by Fisher’s *r*-to-*z* transformation to improve normality.

Similar to the statistical analysis of amygdala–PFC functional connectivities, for each rat, the fALFF and functional connectivity maps were averaged across the 5 sessions. The overall maps for each animal at each of the two ages scanned (adolescence and adulthood) were then forwarded to a mixed-effects ANOVA, with age modeled as a within-subjects factor, and treatment group (Maltreatment vs Control) as a between-subjects factor. The interaction between age and treatment was also modeled. Multiple comparisons across voxels were controlled through Gaussian random field theory correction (voxel level Z>2.3, cluster-level *P*<0.05).

## Results

### Behavioral results

**Figure 1:**
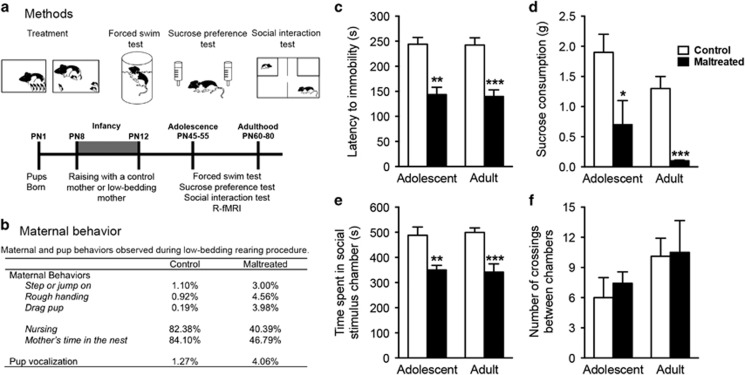
(**a**) Experimental design and timeline. Animals were reared with a caregiver provided with normal or insufficient bedding for nest building, with the latter producing a stressed mother and maltreatment of pups. Following this rearing manipulation, animals were tested in adolescence and adulthood using a test battery associated with depressive-like behavior (forced swim, sucrose preference and social interaction tests). Animals were also scanned twice in both adolescence and adulthood. (**b**) Maltreatment of pups was verified by observations of maternal and pup behaviors during the low bedding procedure. Compared to control mothers, maltreating mothers spent less time inside the nest and nursing pups and handled pups roughly. Behavioral values are expressed as percentage of observation periods in which behaviors occurred for one of the two observation periods. Percentages of behavioral measures do not add to 100% because behaviors can co-occur or not (for example, nursing sometimes occurs outside the nest). (**c**–**f**) Adolescent and adult testing suggests early-life maltreatment produces depressive-like behavior. This affectivity was measured with forced swim (**c**), sucrose preference (**d**), and social interaction (**e**) tests. Differences in social interaction were not due to between group differences in locomotor activity (**f**). Note: *P<0.05, **P<0.01, ***P<0.001. Bars show means and error bars represent s.e.m. R-fMRI, resting-state functional magnetic resonance imaging.

Significant group differences were observed at both adolescence and adulthood on all behavioral tests. Specifically, compared to control rats, maltreated rats reared by a mother with low bedding exhibited reduced latency to stop swimming (adolescents: *t*_(10)_=5.03, *P*=0.002; adults: *t*_(__10)_=5.29, *P*=0.0001), decreased sucrose intake (adolescents: *t*_(__9)_=2.30, *P*=0.04; adults: *t*_(10)_=6.05, *P*=0.0001), and spent less time interacting socially (adolescents: *t*_(12)_=3.71, *P*=0.003; adults: *t*_(12)_=4.49, *P*=0.0007; Figure 1). Notably, these differences were not due to differences in locomotor activity as there were no differences in the number of chamber crossings between groups at both ages (adolescents: *t*_(12)_=0.62, *P*=0.54; adults *t*_(12)_=0.11, *P*=0.91).

### Alterations in developing amygdala–PFC functional connectivity

**Figure 2:**
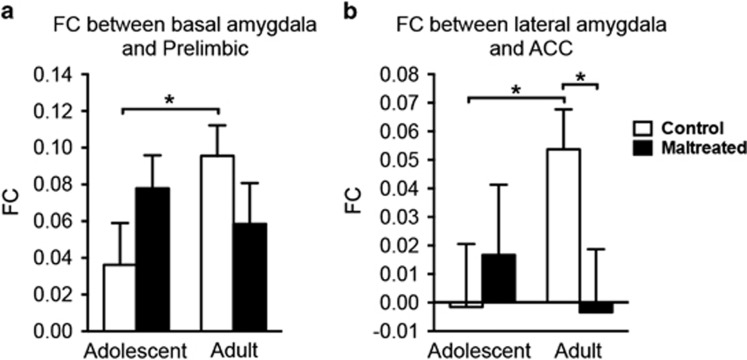
The alterations in amygdala–PFC functional connectivity (FC) development in maltreated (abused) animals. Nearly significant group by age interactions were found on basal amygdala—prelimbic PFC FC (**a**) and lateral amygdala—ACC FC (**b**). In controls, amygdala–PFC FC increased from adolescence to adulthood *(*P*<0.06), whereas maltreated animals did not change significantly. FC between lateral amygdala and ACC was significantly lower in maltreated adult rats than in control adults (P<0.05). Bars show means and error bars represent s.e.m. ACC, anterior cingulate cortex; PFC, prefrontal cortex.

In hypothesis-driven analyses, two pairs of amygdala–PFC functional connectivity edges demonstrated trend group by age interactions (Figure 2; *P*<0.06, uncorrected for nine comparisons). These were: basal amygdala with prelimbic PFC (F_(1,48)_=3.88; *P*=0.055) and lateral amygdala with ACC (F_(1,48)_=3.78; *P*=0.058). We performed further simple effect analyses by comparing adolescence to adulthood for each group with paired t-tests, as well as by comparing the two groups at each time point with two-sample *t*-tests (to verify whether the data meet assumptions for these simple effect *t*-tests, we tested whether the data were normally distributed and whether the variances were equal. For the functional connectivity between basal amygdala and prelimbic PFC, all 4 conditions (maltreated, controls by adolescents and adults) were normally distributed (tested through MATLAB’s Lilliefors test, that is, lillietest), and the variance of the 4 conditions were equal (tested through MATLAB’s multiple-sample tests for equal variances, that is, vartestn). For the functional connectivity between lateral amygdala and ACC, results for one condition were not normally distributed (maltreated adults, *P*=0.005), whereas the variances of all conditions were equal). Contrasts for the functional connectivity between basal amygdala and prelimbic PFC were: control adolescents vs control adults: *t*_(24)_=−1.98, *P*=0.059; maltreated adolescents vs maltreated adults: *t*_(24)_=0.73, *P*=0.47; control adolescents vs maltreated adolescents: *t*_(48)_=−1.43, *P*=0.16; control adults vs maltreated adults: *t*_(48)_=1.34, *P*=0.19. Contrasts for the functional connectivity between lateral amygdala and ACC were: control adolescents vs control adults: *t*_(24)_=−2.00, *P*=0.056; maltreated adolescents vs maltreated adults: *t*_(24)_=−0.55, *P*=0.58; control adolescents vs maltreated adolescents: *t*_(48)_=−1.43, *P*=0.16; control adults vs maltreated adults: *t*_(48)_=2.18, *P*=0.034. In general, amygdala–PFC functional connectivity increased (*P*<0.06) from adolescence to adulthood for controls, while maltreated animals did not change significantly.

### Maltreatment-specific developmental patterns in intrinsic activity

**Figure 3:**
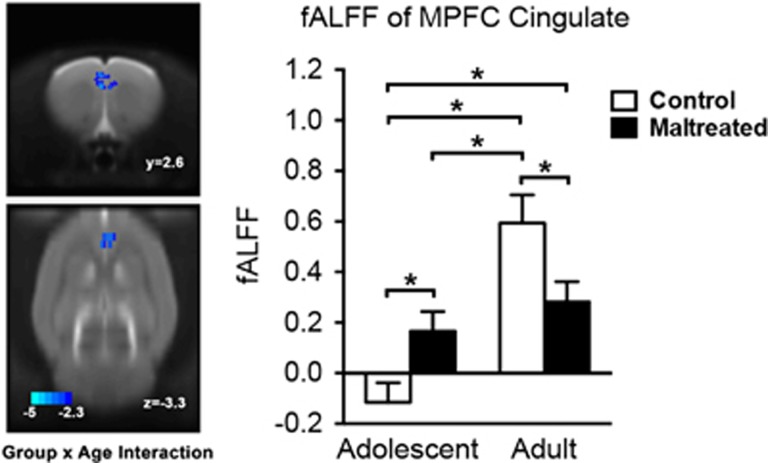
The alterations in developmental trajectories of fractional amplitude of low-frequency fluctuations (fALFF) in medial prefrontal cortex (MPFC) and anterior cingulate cortex (ACC) in maltreated (abused) animals. Intrinsic activity indexed by fALFF in MPFC/ACC increased significantly with age in the control group. By contrast, in the maltreated group, fALFF in MPFC/ACC remained at the same level in adolescence (PN45) and adulthood (PN60). Bars show means and error bars represent s.e.m. *P<0.05.

In exploratory analyses, we found that the groups differed in developmental trajectories of fALFF in MPFC and ACC (Figure 3, Table 1). Intrinsic activity indexed by fALFF in MPFC/ACC increased significantly with age in the control group. By contrast, in the maltreated group, fALFF in MPFC/ACC remained at the same level in adolescence and adulthood. Interestingly, at both ages, the maltreated animals demonstrated significantly higher fALFF than adolescent controls, and significantly lower fALFF than adult controls.

### Maltreatment-related changes in intrinsic functional connectivity

**Figure 4:**
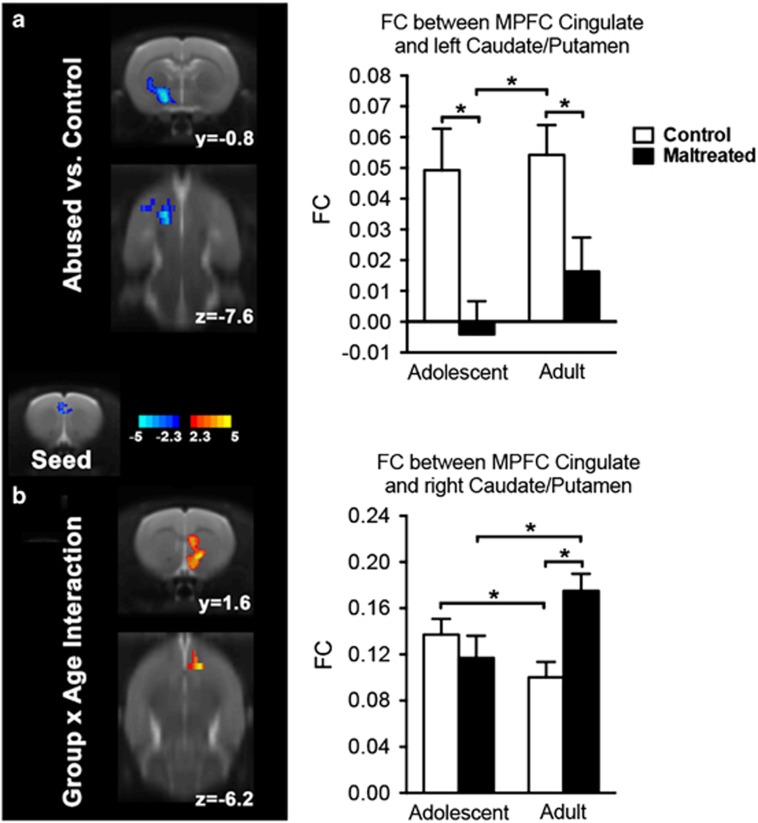
The maltreatment related changes in intrinsic functional connectivity (FC) between medial prefrontal cortex (MPFC)/anterior cingulate cortex (ACC) and left caudate/putamen (**a**) and between MPFC/ACC and right caudate/putamen (**b**). The maltreated group demonstrated reduced FC with MPFC/ACC in left caudate/putamen across both ages (**a**). By contrast, MPFC/ACC FC with right caudate/putamen showed a group by age interaction (**b**). During development, FC with the right caudate/putamen decreased in controls but increased in the maltreated animals. Bars show means and error bars represent s.e.m. *P<0.05.

Building on the significant group by age interaction in fALFF in MPFC/ACC, we defined this cluster as a seed, and explored changes in whole brain functional connectivity. Relative to controls, the maltreated group demonstrated reduced functional connectivity between MPFC/ACC and left caudate/putamen (extending to ventral pallidum, lateral globus pallidus and dorsal endopiriform nucleus) across both ages (Figure 4a, Table 2). By contrast, functional connectivity between MPFC/ACC and right caudate/putamen (extending to nucleus accumbens and corpus callosum) showed a group by age interaction (Figure 4b, Table 3). During development, MPFC/ACC functional connectivity with the right caudate/putamen decreased in controls but increased in the maltreated animals.

## Discussion

Understanding the enduring neurobehavioral effects of infant maltreatment is a high priority for translational developmental neuroscience.^19^ Here we examined this by modulating maternal care through an environmental manipulation (that is, limited bedding) and conducting behavioral assays that measure affective function within an animal model of depressive-like behavior at two subsequent developmental time points. We used R-fMRI to directly translate an approach frequently used in humans, with a longitudinal design and the advantage of full experimental control, which is not attainable in human studies, to help facilitate translation across findings. Consistent with prior work on the neurobiological sequelae of early-life adversity,^9, 13, 14^ infant maltreatment produced long-lasting behavioral effects and altered the development of prefrontal and limbic structures implicated in the pathophysiology of depression.^9, 12, 42, 49^ Emerging research suggests that early-life adversity also impacts functional connectivity between these brain regions.^13, 30, 31, 32^ For example, depriving young children of the sensory stimulation typically experienced by infants during caregiver–infant interactions, as may occur during orphanage rearing, human caregiver maltreatment or nonhuman primates being reared by a maltreating mother, all impact the development of how the PFC and amygdala communicate.^30, 31, 61^ The present work extends these findings by documenting the developmental trajectory of functional connectivity between specific amygdala nuclei and PFC subareas (IL, PL and ACC), which permits closer assessment of our understanding of the diverse functioning of these complex brain areas and their relationship to specific behavioral deficits. This work also extends previous work by expanding the networks involved to brain regions with known anatomical connections to the PFC and amygdala and implicated in affect, such as the striatum (caudate, putamen), nucleus accumbens (core and shell) and corpus callosum.

The maternal maltreatment paradigm used here disrupts mother–infant interactions, as indicated by mothers spending less time with pups and handling them roughly, which resulted in frequent pup distress vocalization (Figure 1b).^9, 46^ Previous work demonstrates this procedure has enduring effects resulting in a later-life depressive-like phenotype and aberrant amygdala and PFC activity, although these deficits do not emerge until after weaning.^1, 9, 46^ In accordance with those prior reports, rat pups reared by maltreating mothers displayed depressive-like behaviors, including performance on the forced swim, sucrose preference and social behavior tests (Figure 1). This rodent model of maltreatment targets pups during the sensitive period for attachment to the caregiver and overlaps with functional development of the amygdala^1, 43^ but prior to functional development of the striatum^62^ and the PFC.^63^ These results parallel the maltreatment literature in children: abuse is most frequent between birth and 3 years of age and produces neurobiological sequelae including social withdrawal, depression-like symptomatology, anhedonia, atypical amygdala activity and altered trajectories of brain development.^5, 6, 15, 23, 64^

We found that functional connectivity between basal amygdala and prelimbic PFC increased in control animals, as did the functional connectivity between lateral amygdala and ACC. (The non-negative FC between PFC and amygdala is consistent with a prior result that anti-correlations were absent in anesthetized rats even with global signal regression^65^). This is in line with a cross-sectional study in humans, which investigated the normative developmental emergence of functional connectivity between amygdala and MPFC and found an age-related increase.^66^ Studies proposing dual-system models suggest that amygdala functioning is down-regulated by MPFC, and that such top–down regulation increases with age.^67, 68, 69^ Interestingly, in maltreated animals, we found that amygdala-MPFC functional connectivity failed to develop (that is, it did not differ significantly between adolescence and adulthood). This is consistent with the finding that patients with major depressive disorder and a history of childhood maltreatment exhibit widespread reductions in intrinsic connectivity of prefrontal-limbic regions compared to controls.^70^ Moreover, in a cross-sectional task-based functional connectivity study in humans, Gee et al.^13^ reported significant age-related effects on amygdala-MPFC functional connectivity in normal controls but not in individuals with a history of orphanage rearing. Despite the many differences in study design, including scanning animals under anesthesia, the consistent observation of failed development in MPFC regulatory effects on amygdala after maternal maltreatment suggests they may underlie depressive-like behaviors later in life in humans as they did in our animals. As rodent and human MPFC differ somewhat anatomically, we note that the MPFC of the rodent is comprised of IL and PL, whereas the MPFC in humans overlaps with the ACC.^71^

An exploratory analysis allowed us to examine developmental alterations induced by maltreatment on the strength of intrinsic brain activity (indexed by fALFF) across the brain. The fALFF in prelimbic MPFC and ACC significantly increased during development in controls from adolescence to adulthood. However, maltreated animals already showed significantly higher fALFF than controls in adolescence, that is, maltreatment accelerated the development of intrinsic brain activity strength in MPFC/ACC. This is consistent with early maturation or accelerated development models of early-life adversity,^41^ that is, maternal deprivation accelerates the development of intrinsic brain activity, which has been demonstrated in humans.^13^ Yet, the intrinsic brain activity strength of the maltreated group in MPFC/ACC failed to reach the level of the controls in adulthood. The MPFC and ACC have been implicated in emotional regulation^8^ by exerting top–down inhibitory control over amygdala reactivity to dampen emotional responding.^72^ Hence, failed development in MPFC/ACC intrinsic activity may contribute to compromised development in emotional regulation circuits, resulting in later depressive-like behaviors in both rodents and humans. Indeed, maltreated animals exhibit increased depressive-like behaviors (that is, increased behavioral despair, social withdrawal and anhedonia) and amygdala hyperactivity in response to stress compared with controls,^9, 12^ which is consistent with impaired bottom-up signaling for top–down cortical modulation of limbic regions regulating affective behaviors.

Follow-up analysis of the MPFC/ACC as a seed revealed that the functional connectivity between MPFC/ACC and left dorsal striatum (caudate/putamen) demonstrated significant group differences between the maltreated and control groups, at both ages tested. The prelimbic MPFC has dense projections to the dorsal striatum,^12, 73^ and those projections are related to reward processing.^74^ Reduced MPFC-dorsal striatum functional connectivity in maltreated animals suggests that impaired reward processing may underlie the depressive-like behaviors in maltreated rats, for example, the lower consumption of sucrose. In humans, exposure to early-life stress is associated with blunted reward-related striatal activity and increased depression-like symptomatology in adolescence and adulthood.^75, 76, 77^ Furthermore, neuroimaging studies have reported decreased reward-related activity in depressed patients, thereby establishing a link between dysfunction of the positive valence system and depression.^78, 79^ Collectively, these data suggest early-life stress induces alterations in the neural circuitry that supports reward processing, which may underlie the emergence of depression-like symptomatology.

Although functional connectivity differences between MPFC/ACC and the left dorsal striatum were observed between maltreated and control groups at both ages, we observed a partial shifting of functional connectivity in control and maltreated animals in the right dorsal striatum. Both groups of animals showed stronger right hemisphere striatal functional connectivity. However, in controls, this decreased with age, whereas in maltreated animals, it became stronger with age. A similar lateralization is observed in humans. Individuals exposed to maltreatment report elevated depressive symptoms and show decreased anticipatory activity in the left basal ganglia compared to control subjects.^80^ Thus, early-life adversity is associated with lateralized dysfunction within the MPFC/ACC and the striatum, and this dysfunction may serve as a diathesis contributing to the multiple negative outcomes stemming from maltreatment. Indeed, humans appear to exhibit a lateralization for affective processes, with positive affect preferentially represented in left-sided prefrontal territories and negative affect preferentially represented in right-sided prefrontal areas.^81^ Within this context, our findings of reduced left prefrontal–striatum functional connectivity and increased right prefrontal–striatum functional connectivity are consistent with a reduction in positive affect and an increase in negative affect, respectively.

Several limitations should be noted. First, the use of anesthesia inevitably affects functional connectivity.^65^ Second, our study was conducted entirely in males, so results may not generalize to females. Third, the behavioral tests and MRI examination were performed on separate samples to avoid the possible confounding impact of behavioral testing on brain networks. Thus, we are not able to examine the association between fMRI connectivity/fALFF measures and behavioral performance. Fourth, we did not perform evaluations in infancy, thus we could not evaluate the full development trajectory from infancy to adulthood. These are balanced by study strengths of providing the first longitudinal examinations in developing rodents, within the context of an ecologically valid environmental manipulation.

## Conclusions

This study provides a translational link between human and animal model research on early-life stress by using R-fMRI, a commonly used technique in humans that can be applied to rodents to provide a common data point between species. We used a naturalistic rodent model of maternal maltreatment that results in a later-life depressive-like phenotype to model early-life trauma within the attachment system in children. Our data not only show convergence with human data on orphanage-reared children but also with nonhuman primate data. We confirmed that intrinsic functional connectivity exhibits robust developmental effects that parallel known trajectories of synaptic connectivity in humans and provide evidence that caregiver maltreatment during infancy results in differences in brain functional connectivity in later-life that likely underlie negative affectivity and vulnerability to psychopathology. Thus, enhanced susceptibility to psychopathology following early-life adversity is associated with differential developmental trajectories of prefrontal and subcortical circuits, particularly those involving the MPFC/ACC.

## Figures and Tables

**Table 1:** Cluster information of significant age by group (treatment) interaction on fALFF

	*Paxinos area number*	*Paxinos area name*	*Cluster volume (mm*^*3*^)
Whole cluster^a^	691/212	MPFC/Cingulate	2.75
Subarea 1	691	Prelimbic cortex	1.29
Subarea 2	212	Cingulate cortex, area 1	1.26
Subarea 3	—	Not defined	0.20

Abbreviations: fALFF, fractional amplitude of low-frequency fluctuations; MPFC, medial prefrontal cortex.

a Note: the peak *T* value of the whole cluster is −3.79 at coordinates: *x*=−0.4, *y*=2.4, *z*=−3.1.

**Table 2:** Significantly different clusters of intrinsic functional connectivity with MPFC/Cingulate (fALFF defined seed)

	*Paxinos area number*	*Paxinos area name*	*Cluster volume (mm*^*3*^)
Whole cluster^a^	232/919/458/254/1015/259/416/80	Left caudate putamen	16.53
Subarea 1	232	Caudate putamen (striatum)	6.82
Subarea 2	919	Ventral pallidum	0.98
Subarea 3	458	Lateral globus pallidus	0.78
Subarea 4	254	Dorsal endopiriform nucleus	0.74
Subarea 5	1015	Piriform layer	0.51
Subarea 6	259	Dysgranular insular cortex	0.37
Subarea 7	416	Interstitial nucleus of the posterior limb of the anterior commissure	0.37
Subarea 8	80	Accumbens nucleus, shell	0.34
Subarea 9	—	Not defined	5.60

Abbreviations: fALFF, fractional amplitude of low-frequency fluctuations; MPFC, medial prefrontal cortex.

a Note: the peak *T* value of the whole cluster is −4.08 at coordinates: *x*=−2.3; *y*=−0.8; *z*=−7.6.

**Table 3:** Cluster information of significant age by treatment group interaction on intrinsic functional connectivity with MPFC/Cingulate (fALFF defined seed)

	*Paxinos area number*	*Paxinos area name*	*Cluster volume (mm*^*3*^)
Whole cluster^a^	232/79/200 80/330/691	Right caudate putamen	9.30
Subarea 1	232	Caudate putamen (striatum)	4.11
Subarea 2	79	Accumbens nucleus, core	1.49
Subarea 3	200	Corpus callosum	0.61
Subarea 4	80	Accumbens nucleus, shell	0.54
Subarea 5	330	Forceps minor of the corpus callosum	0.51
Subarea 6	691	Prelimbic cortex	0.37
Subarea 7	0	Not defined	1.66

Abbreviations: fALFF, fractional amplitude of low-frequency fluctuations; MPFC, medial prefrontal cortex.

a Note: the peak *T* value of the whole cluster is 3.68 at coordinates: *x*=1.8; y=1.6; z=−6.2.
